# *OTUD5* Variants Associated With X-Linked Intellectual Disability and Congenital Malformation

**DOI:** 10.3389/fcell.2021.631428

**Published:** 2021-03-03

**Authors:** Ken Saida, Tokiko Fukuda, Daryl A. Scott, Toru Sengoku, Kazuhiro Ogata, Annarita Nicosia, Andres Hernandez-Garcia, Seema R. Lalani, Mahshid S. Azamian, Haley Streff, Pengfei Liu, Hongzheng Dai, Takeshi Mizuguchi, Satoko Miyatake, Miki Asahina, Tsutomu Ogata, Noriko Miyake, Naomichi Matsumoto

**Affiliations:** ^1^Department of Human Genetics, Yokohama City University Graduate School of Medicine, Yokohama, Japan; ^2^Department of Pediatrics, Hamamatsu University School of Medicine, Hamamatsu, Japan; ^3^Department of Molecular and Human Genetics, Baylor College of Medicine, Houston, TX, United States; ^4^Texas Children’s Hospital, Houston, TX, United States; ^5^Department of Molecular Physiology and Biophysics, Baylor College of Medicine, Houston, TX, United States; ^6^Department of Biochemistry, Yokohama City University Graduate School of Medicine, Yokohama, Japan; ^7^Baylor Genetics, Houston, TX, United States; ^8^Department of Pediatrics, Hamamatsu City Welfare and Medical Center for Development, Hamamatsu, Japan

**Keywords:** OTUD5, X-linked intellectual disability, LINKED syndrome, deubiquitinase, congenital malformation

## Abstract

**Background:**

X-linked intellectual disability (XLID), which occurs predominantly in males, is a relatively common and genetically heterogeneous disorder in which over 100 mutated genes have been reported. The *OTUD5* gene at Xp11.23 encodes ovarian tumor deubiquitinase 5 protein, which is a deubiquitinating enzyme member of the ovarian tumor family. LINKage-specific-deubiquitylation-deficiency-induced embryonic defects (LINKED) syndrome, arising from pathogenic *OTUD5* variants, was recently reported as a new XLID with additional congenital anomalies.

**Methods:**

We investigated three affected males (49- and 47-year-old brothers [Individuals 1 and 2] and a 2-year-old boy [Individual 3]) from two families who showed developmental delay. Their common clinical features included developmental delay, hypotonia, short stature, and distinctive facial features, such as telecanthus and a depressed nasal bridge. Individuals 1 and 2 showed epilepsy and brain magnetic resonance imaging showed a thin corpus callosum and mild ventriculomegaly. Individual 3 showed congenital malformations, including tetralogy of Fallot, hypospadias, and bilateral cryptorchidism. To identify the genetic cause of these features, we performed whole-exome sequencing.

**Results:**

A hemizygous *OTUD5* missense variant, c.878A>T, p.Asn293Ile [NM_017602.4], was identified in one family with Individuals 1 and 2, and another missense variant, c.1210 C>T, p.Arg404Trp, in the other family with Individual 3, respectively. The former variant has not been registered in public databases and was predicted to be pathogenic by multiple *in silico* prediction tools. The latter variant p.Arg404Trp was previously reported as a pathogenic *OTUD5* variant, and Individual 3 showed a typical LINKED syndrome phenotype. However, Individuals 1 and 2, with the novel variant (p.Asn293Ile), showed no cardiac or genitourinary malformations.

**Conclusions:**

Unlike previous reports of LINKED syndrome, which described early lethality with congenital cardiac anomalies, our three cases are still alive. Notably, the adult brothers with the novel missense *OTUD5* variant have lived into their forties. This may be indicative of a milder phenotype as a possible genotype-phenotype correlation. These findings imply a possible long-term prognosis for individuals with this new XLID syndrome, and a wider phenotypic variation than initially thought.

## Introduction

X-linked intellectual disability (XLID) is a relatively common disorder that is predominantly observed in males. XLID is genetically heterogeneous, and is caused by more than 100 protein-coding genes in the X chromosome (Muthusamy et al., 2017; Neri et al., 2018). Aberrations in these X-linked genes are estimated to account for 5–10% of male patient with intellectual disability (Lubs et al., 2012; Tzschach et al., 2015; Muthusamy et al., 2017). Next-generation sequencing has led to a marked increase in identification of disease-causing variants in many new and previously unresolved cases of familial and sporadic genetic disorders (Muthusamy et al., 2017; Neri et al., 2018).

The *OTUD5* gene (MIM#300713) at Xp11.23 encodes ovarian tumor (OTU) deubiquitinase 5 protein, which is a deubiquitinating enzymes (DUBs) member of the OTU family (Mevissen et al., 2013). DUBs are proteases that specifically cleave ubiquitin linkages. There are approximately 100 DUBs, including OTUD5, in humans (Komander et al., 2009; Mevissen et al., 2013). OTU DUBs are important regulators of the ubiquitin code, and therefore, they control crucial physiological processes in humans (Kulathu and Komander, 2012; Mevissen et al., 2013). Abnormalities in genes encoding OTU DUBs cause various developmental or autoinflammatorydiseases (Basar et al., 2020). Recently, a male-specific multiple congenital anomaly disorder caused by pathogenic variants in *OTUD5* was reported by two groups (Beck et al., 2021; Tripolszki et al., 2021). Tripolszki et al. described a large family with 13 affected individuals (Tripolszki et al., 2021). Beck et al. independently reported ten pediatric cases from seven different families with an *OTUD5* pathogenic variant and named this disease LINKage-specific-deubiquitylation-deficiency-induced embryonic defects (LINKED) syndrome (Beck et al., 2021). Affected individuals were characterized by global developmental delay, congenital cardiac anomalies, genitourinary abnormalities, and dysmorphic faces.

In the present study, we used whole-exome sequencing (WES) to identify pathogenic hemizygous variants in three individuals from two families with suspected XLID.

## Materials and Methods

### Subjects

**FIGURE 1:**
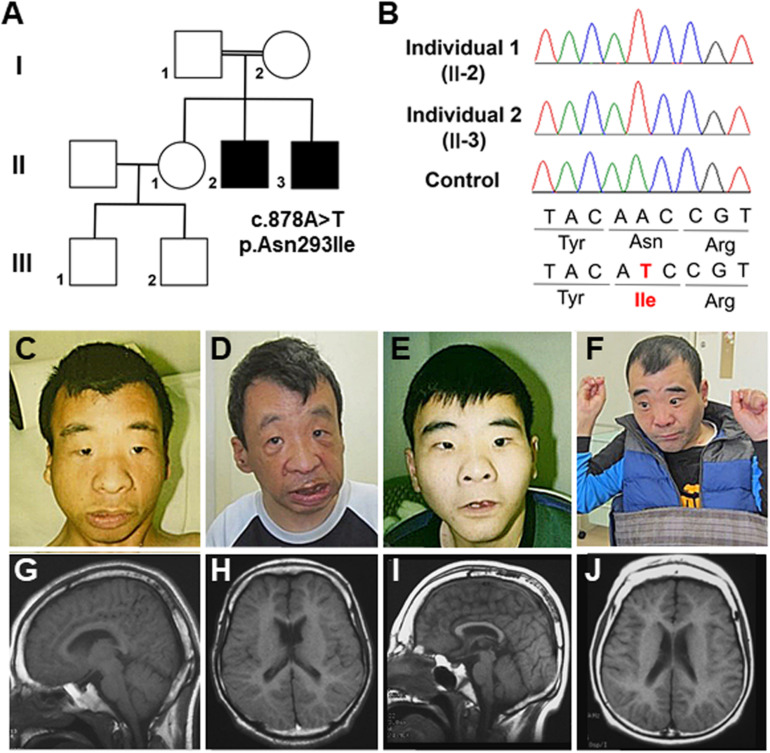
**(A)** Familial pedigree of the affected male siblings. **(B)** Electropherograms of the *OTUD5* variant (c.878A>T, p.Asn293Ile [NM_017602.4]) in the affected brothers and the control. **(C,D)** Photographs of the older brother (II-2) at 22 and 48 years of age. **(E,F)** Photographs of the younger brother (II-3) at 20 and 46 years of age. **(G,H)** Brain magnetic resonance images (MRI). Sagittal and coronal views of T1 susceptibility-weighted images of the older brother at 24 years of age are shown. A thin corpus callosum, a small frontal lobe, and volume loss of the subcortical white matter and cavity of the septum pellucidum can be seen. **(I,J)** Brain MRI. Sagittal and coronal views of T1 susceptibility-weighted (SW) images of the younger brother at 22 years of age are shown. A thin corpus callosum, frontal lobe hypoplasia, volume loss of the subcortical white matter, colpocephaly, and hypoplasia of the cerebellar hemispheres can be seen.

**FIGURE 2:**
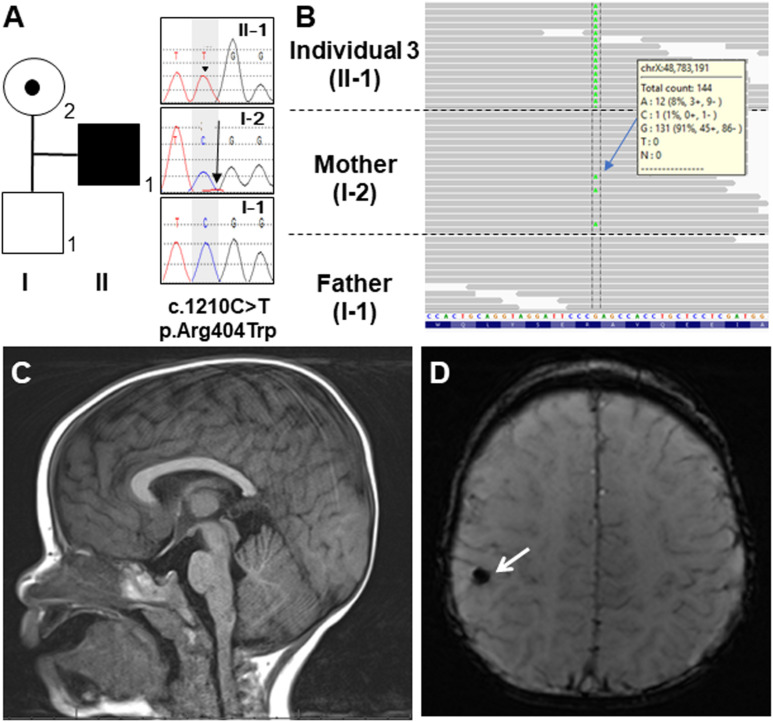
**(A)** Familial pedigree of Individual 3 and DNA sequence electropherograms illustrating the *OTUD5* variant (c.1210C>T, p.Asn404Trp [NM_017602.4]). Arrow head of the proband (II-1) in the electropherogram shows the mutated allele (C>T) in a hemizygous state. The arrow in the mother’s electropherogram (I-2) indicates the mutated allele in a mosaic state. **(B)** Integrated genomic view demonstrating that the variant can be seen in the hemizygous state in Individual 3 and in some of the reads from his mosaic mother. **(C)** Sagittal view of the brain MRI of Individual 3 at 19 months of age. His corpus callosum was normal. **(D)** Coronal view. The white arrow showed a hypointense focus in the right perirolandic region, which most likely represents hemosiderin deposition secondary to a previous microhemorrhage.

We used the GeneMatcher (Sobreira et al., 2015) system to connect two centers at Yokohama City University Graduate School of Medicine (Yokohama, Japan) and Baylor College of Medicine (Houston, TX, United States). Written informed consent was obtained from the patients’ or their parents based on experimental protocols, approved by the Institutional Review Board of each center. Affected Individuals 1 and 2 were 49- and 47-year-old Japanese brothers (Figure 1A; II-2 and II-3) born to healthy consanguineous parents (the parents were second cousins). These patients had an unaffected sister (Figure 1A; II-1). Genomic DNA samples were extracted from peripheral blood leukocytes of the affected brothers and an unrelated control using a QIAamp DNA Mini Kit (Qiagen, Hilden, Germany). Individual 3 (Figure 2A; II-1) was a 2-year-old boy of European descent. He was the first child born to non-consanguineous parents (Figure 2A; I-1,2). The family history was non-contributory.

### WES and Protein Structure Analysis

For Individuals 1 and 2, WES was performed using an illumina platform as previously described (Miyake et al., 2020). The mean read depth of the RefSeq coding region was 98.3 and 113.6 reads (corresponding to 96.1%- and 96.4%-, respectively, covered by >20 reads), respectively. Analyses based on autosomal dominant (*de novo*), autosomal recessive (homozygous and compound heterozygous), and X-linked models were conducted. For X-linked recessive conditions, we picked up candidate genetic variants in exons and canonical splice sites (±2 bp) with a minor allele frequency of <0.005 using the Exome Aggregation Consortium browser (ExAC), NHLBI Exome Variant Server (ESP6500), Human Gene Mutation Database (HGMD) and in-house WES Japanese control data (*n* = 575). Candidate variants were prioritized based on the biological and clinical relevance of each gene to the phenotype of the patients. Potential candidate variants were validated using the Sanger method, using an ABI3500xL sequencer (Applied Biosystems, Foster City, CA, United States) and Sequencher 5.0 (Gene Codes Corporation, Ann Arbor, MI, United States). Structural analysis and figure preparation of the OTUD5 protein were performed using PyMOL (Schrödinger, Inc., New York, NY, United States).

For Individual 3, array-based copy number variant (CNV) analysis (CMA-HR + SNP version 11.2) and trio WES were performed on a clinical basis at Baylor Genetics^1^ (Yang et al., 2013, 2014; Wiszniewska et al., 2014). The following quality control metrics for exome sequencing are generally achieved: >70% of reads are aligned to the target with >95% of targeted bases covered at >20 reads, >85% of targeted bases are covered at >40 reads, and mean coverage of targeted bases is >100 reads.

## Results

### Clinical Findings

Individual 1, the older brother (II-2), was born at 42 weeks of gestational age by spontaneous vaginal delivery. His birth weight, length, and head circumference were 2,700 g [–0.6 standard deviation (SD)], 46.5 cm (–1.2 SD), and 31.5 cm (–1.3 SD), respectively. At 1 month of age, he appeared cyanotic directly after feeding; however, echocardiography revealed no cardiac abnormalities. To prevent cyanosis, gastric tube feeding was administered for several months. His developmental milestones were delayed. He had head control at 6 months, crawled at 30 months, and walked independently at 4 years. He spoke no meaningful words and gained no language comprehension. Dysmorphic features, including thick eyebrows, telecanthus, widely spaced eyes, down-slanted palpebral fissure, a wide and depressed nasal bridge, a wide nasal base, thin upper lip, and thick lower lip, have been noted since his infancy (Figures 1C,D). At 4 years of age, he experienced generalized tonic–clonic seizures, and his electroencephalogram showed paroxysmal discharges. Therefore, he started medication with phenobarbital and carbamazepine; however, his seizures continued once a month until adolescence. At 22 years of age, his height was 142 cm (–5.0 SD) and his weight was 41.0 kg (–2.1 SD). He was able to walk independently, although his gait was short-stepped with arm flexion and knee extension. During his most recent evaluation, at 48 years of age, he was still able to walk independently and his epileptic seizures were controlled with carbamazepine and zonisamide; however, he had intellectual disability.

Individual 2 (II-3) is the younger brother of Individual 1. He was born at 39 weeks of gestational age by spontaneous vaginal delivery, with no complications. His birth weight, length, and head circumference were 2,600 g (–1.4 SD), 46.0 cm (–1.0 SD), and 31.0 cm (–1.6 SD), respectively. He showed significant phenotypic overlap with Individual 1, including the dysmorphic features (Figures 1E,F) and delayed developmental milestones; he had head control at 7 months, sat with support at 22 months, and crawled at 24 months. He was able to stand with support at 4 years, but was never able to walk. He spoke no meaningful words and gained no language comprehension. At 1 year of age, he also began to experience generalized tonic–clonic seizures. Although he was treated with valproate, clonazepam, and carbamazepine, drop attacks occurred once every few months. At 20 years of age, his height was 134.3 cm (–6.3 SD) and his weight was 37.1kg (–2.5 SD). He was able to stand with support, with flexed knee joints and ankle valgus. During his most recent evaluation, at 46 years of age, he was unable to stand alone and had intellectual disability. He usually sat with his shoulder joints flexed forward (at approximately 80°), flexed elbow joints, forearm pronation, and with his palms grasped softly. A physical examination showed no joint contracture. Left cryptorchidism was noted. Monthly epileptic drop seizures remained, even under medication with carbamazepine, valproate, and clonazepam.

Brain magnetic resonance imaging (MRI) of Individual 1 at the age of 24 years and of Individual 2 at the age of 22 years revealed a thin corpus callosum or colpocephaly, a small frontal lobe, and volume loss of the subcortical white matter (Figures 1G–J). Ultrasonographic examination of the heart and kidney was normal. G-banded chromosome analysis revealed normal karyotypes (46, XY) in both affected brothers. Array comparative genomic hybridization analysis indicated a duplication of the 5q31.1 region (a region of chr5:130551687–130740870 in Individual 1 and chr5:130484163–130731837 in Individual 2, based on hg19) (Asahina et al., 2016). However, there have been few reports of individuals with 5q31.1 duplications, and a characteristic phenotype is not obvious (Rosenfeld et al., 2011). Moreover, our patients did not share many aspects of the phenotypes of other individuals with 5q31.1 duplications. Therefore, the pathogenic significance of the 5q31.1 duplication in our patients remains unknown.

Individual 3 is a 2-year-5-month boy with no history of prenatal teratogenic exposure. A 20-week ultrasound revealed hyperechoic areas in the brain and heart. These areas were not identified on subsequent ultrasound examinations, but unilateral, renal hyperechogenicity with pelviectasis was observed. Individual 3 was delivered at 40 weeks’ gestation via cesarean section because of failure to progress. After birth, he was found to have a cardiac murmur, penoscrotal hypospadias, bilateral cryptorchidism, bilateral inguinal hernias, ankyloglossia, and a high arched palate. An echocardiogram revealed tetralogy of Fallot (TOF) with valvular and subvalvular pulmonic stenosis. A post-natal renal ultrasound was normal. He was discharged at one week of age. His TOF was repaired at 7 months of age, and he had bilateral ochiopexy and inguinal hernia repair at 11 months of age.

At 2 months of age, Individual 3 was noted to have feeding difficulties and failure to thrive. He was diagnosed with gastroesophageal reflux disease (GERD) at 3 months of age. This diagnosis was confirmed during a videofluoroscopic swallow study performed at age 7 months that also revealed esophageal dysmotility and minimal laryngeal vestibular penetration with thin liquids. This was treated by using a different artificial nipple for feeds. His GERD is currently being treated with lansoprazole. Over time, global developmental delay and diffuse hypotonia were also observed. He rolled over at 5–7 months of age and sat at 6 months of age but is still unable to walk independently and he does not use any words or signs. Brain MRI at 19 months of age was normal except for a punctate T2 hypointense focus in the right perirolandic region with corresponding blooming on susceptibility-weighted imaging (SWI) that most likely represents hemosiderin deposition secondary to a previous microhemorrhage (Figures 2C,D).

A skeletal survey obtained at 18 months of age due to short stature and abnormal, circumferential fat distribution over the extremities was normal. However, at 19 months of age he was diagnosed with growth hormone deficiency based on the finding of undetectable insulin-like growth factor-1 and insulin-like growth factor binding protein-3 levels and bilateral pseudopapilledema (Collett-Solberg et al., 1998). He was treated with growth hormone starting at 20 months of age. After starting growth hormone treatment, his abnormal fat distribution resolved and his parents noted an increased appetite, activity and engagement in therapies. However, there was no appreciable change in his height velocity or weight. At 27 months of age his length was 74.4 cm (<0.01 centile, Z = −4.46) and his weight was 8.15 kg (<0.01 centile, Z = −4.76).

Individual 3’s last comprehensive physical examination was at 19 months of age. He had brachycephaly, a prominent forehead, deep set eyes, intermittent bilateral esotropia, and a short nose with a flat nasal root and bulbous tip. He had a left-sided supernumerary nipple and penoscrotal hypospadias. His height was 69.3 cm (<0.01 centile, Z = −4.48), his weight was 8 kg (<0.01 centile, Z = −3.82), and his head circumference was 46.5 cm (13th centile). Array-based copy number variant analysis was normal.

### Identification of the Pathogenic Variant

**FIGURE 3:**
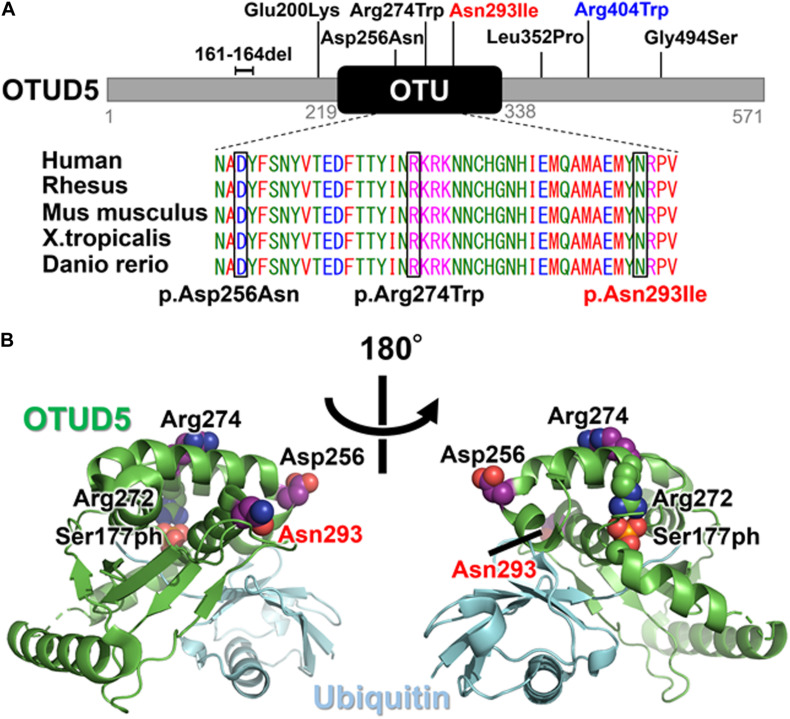
**(A)** Schematic presentation of the OTUD5 protein and the pathogenic variants. The affected amino acid in the OTU domain is conserved among several species, from humans to fish. The protein sequences of the different species were aligned using Clustal Omega. The previously reported variants are in black and the novel variant (Asn293Ile) is shown in red above the protein. The recurrent variant (Arg404Trp) identified in this study and reported by Beck et al. is shown in blue. **(B)** The crystal structure of the Ser177-phosphorylated form of OTUD5 bound with ubiquitin (PDB ID: 3TMP). OTUD5 and ubiquitin are shown in green and cyan, respectively. The pathogenic variant positions (Asp256, Arg274, and Asn293) are indicated.

By searching for candidate variants shared in both of the affected Individuals 1 and 2, and comparing their phenotypes with the variant’s relevance, we identified missense variants in *OTUD5* (c.878A>T, p.Asn293Ile [NM_017602.4]) and *ATP2B3* (c.2768G>A, p.Arg923His [NM_001001344.2]), both located on the X chromosome. Sanger sequencing confirmed that the *OTUD5* and *ATP2B3* variants were hemizygous in both affected siblings. Unfortunately, parental samples could not be obtained. Because the *ATP2B3* variant (c.2768G>A, p.Arg923His, rs201753621) was found in the Genome Aggregation Database (gnomAD) in the hemizygous state (3 of 183,343 alleles), we felt that it was unlikely to be causative. The *OTUD5* variant was absent from all searched public databases (gnomAD, ExAC, ESP6500, and HGMD) and our in-house control database. Moreover, according to the gnomAD database, *OTUD5* is intolerant to amino acid substitutions and loss-of-function alleles (0 observed vs. 17.2 expected, loss-of-function intolerance score: 1.0); the missense intolerance (Z) score is 3.99 (44 observed single nucleotide variants vs. 204.9 expected single nucleotide variants). The altered amino residue in our patients was located on the OTU domain and is highly conserved in multiple species (Figure 3A). Web-based *in silico* software predicted this variant to be pathogenic, with a predicted value of 0.002 (deleterious) in SIFT, 0.999 (probably damaging) in PolyPhen-2, and 1 (disease-causing) in MutationTaster, and a combined annotation dependent depletion score (CADD) of 27.7 (>20). In the crystal structure of OTUD5 bound with ubiquitin (Huang et al., 2012), Asp256, Arg274, and Asn293 are located on the surface of the protein, and form no direct interactions with ubiquitin (Figure 3B). We used IntarVar to evaluate the variant (Li and Wang, 2017), and the results showed that this variant (c.878A>T, p.Asn293Ile) is currently considered a “variant of unknown significance” (VUS, PM1, PM2, and PP3) based on ACMG-AMP guidelines (Richards et al., 2015).

In Individual 3, trio exome sequencing performed on a clinical basis revealed a hemizygous c.1210 C>T, p.Arg404Trp variant in *OTUD5*. This variant was inherited from his mother who was mosaic for the variant (Figures 2A,B). The arginine at position 404 in *OTUD5* is highly conserved. This change was predicted to be deleterious by SIFT, probably damaging by PolyPhen2, disease causing by MutationTaster and has a CADD score of 33 as shown in Table 1. This variant was classified as “likely pathogenic” (PS1, PM2, PP3, and PP4) based on ACMG-AMP guidelines (Richards et al., 2015).

**TABLE 1:** Evaluation of the *OTUD5* variants identified in this study.

*OTUD*5 variant [NM_017602.4]	CytoBand	Position	gnomAD	SIFT score	PolyPhen-2	MutationTaster	CADD score	Novel/Reported
c.878A>T, p.Asn293Ile	Xp11.23	48792016	None	0.002 Deleterious	0.999 Probably damaging	1 Disease causing	27.7 (>20)	Novel
c.1210C>T, p.Arg404Trp	Xp11.23	48783191	None	0 Deleterious	0.997 Probably damaging	1 Disease causing	33 (>20)	Reported (Beck et al., 2021)

*gnomAD, Genome Aggregation Database; CADD, combined annotation dependent depletion.*

## Discussion

In the present study, three affected individuals with *OTUD5* missense variants were identified who had short stature and developmental delay with distinctive facial features. Individuals 1 and 2 were male siblings and had a novel *OTUD5* variant (c.878A>T, Asn293Ile) and sporadic Individual 3 had a known *OTUD5* variant (c.1210C>T, Arg404Trp). Including our patients, almost all of the identified *OTUD5* variants in affected individuals have been missense variants (Beck et al., 2021; Tripolszki et al., 2021).

The high loss-of-function intolerance score and the especially high Z score (3.99, within the top 3% of genes on the X chromosome) for *OTUD5* that were obtained from intolerance tests in the gnomAD database strongly suggest that this gene, with its variants, could be a strong candidate for causing X-linked disorders in humans. The *OTUD5* gene is ubiquitously expressed in human tissues. OTUD5 is an essential component for chromatin remodeling of gene regulatory elements and plays an important role during embryogenesis (Beck et al., 2021).

The novel variant that was identified in the current study (c.878A>T, p.Asn293Ile) is located in the OTU domain, which confers deubiquitinase activity. Within the OTU domain, two other pathogenic variants (c.766G>A, p.Asp256Asn and c.820C>T, p.Arg274Trp) have previously been reported (Figure 3A; Beck et al., 2021). The catalytic activity of OTUD5 is activated when Ser177 is phosphorylated (Huang et al., 2012). In the crystal structure of the Ser177-phosphorylated form of OTUD5 bound with ubiquitin (Huang et al., 2012), the phosphate group has intramolecular interactions with other OTUD5 polar residues (including Arg272), and also has intermolecular interactions with the C-terminal tail of the bound ubiquitin, precisely orienting its C-terminus for catalysis. The pathogenic residues (Asp256, Arg274, and Asn293) are all mapped onto the surface of the protein, suggesting that the variant proteins may not have compromised folding activity. Furthermore, these residues form no direct interactions with either the bound ubiquitin or the phosphate group of Ser177. However, these three residues are all located on the same side of OTUD5. Therefore, these residues might cooperatively mediate interactions with some protein factors that regulate the cellular function of OTUD5, such as the Ser177 kinase. Alternatively, because Arg274 is located close to Arg272, which is one of the critical residues for phosphorylation-dependent activation, it may be involved in structural transition upon Ser177 phosphorylation.

In Individual 3, the known pathogenic variant (c.1210C>T, Arg404Trp) was inherited from his mother who was mosaic. The *OTUD5* variant identified in Individuals 1 and 2 was assumed to be inherited from their mother, but unfortunately a maternal sample was unavailable for this study. Tripolszki et al. reported that females carriers were asymptomatic and X-inactivation study showed 100% inactivation of the maternally inherited X chromosome in four female carriers (Tripolszki et al., 2021).

Phenotypically, early lethality was described in both previous reports associated with *OTUD5* pathogenic variants (Beck et al., 2021; Tripolszki et al., 2021). Causes of death included congenital cardiac complications or sepsis. A clinical summary of the previous studies and the three cases from the present study is shown in Table 2. Our three affected individuals are still alive. Individual 3, who had the known variant p. Arg404Trp, displayed a similar phenotype as that in a previous report, with congenital anomalies such as hypospadias and heart defects, whereas no obvious structural abnormalities in the brain, nor seizures were observed. Individuals 1 and 2, who had the novel variant p.Asn293Ile, showed a relatively milder phenotype. Both affected brothers had severe short stature and refractory epilepsy, even while taking anti-epileptic drugs. Unlike the patients in the previous reports, Individuals 1 and 2 did not show any congenital cardiac anomalies or genitourinary anomalies and have lived to almost 50 years old, suggesting that cardiac involvement may be associated with poor prognosis.

**TABLE 2:** Clinical features of current and reported patients with LINKED syndrome.

Clinical features	Beck et al., 2021	Tripolszki et al., 2021	Present study			Total
	P1-10 (*n* = 10)	VI-2, 6, V-4, 6, 7, 11, IV-3, 4, 5, 9, 10, 11, 16 (*n* = 13)	Individual 1	Individual 2	Individual 3	
*OTUD5* variant	c.482_490del, c.766G>A, c.820C>T, c.1055 T>C, c.1210C>T, c.1480 G>A	c.598G>A	c.878A>T		c.1210C>T	
Protein change	p.161_164del, p.Asp256Asn, p.Arg274Trp, Leu352Pro, p.Arg404Trp, p.Gly494Ser	p.Glu200Lys	p.Asn293Ile		p.Arg404Trp	
Age (years)	2–14 y	4 days–37 y	49 y	47 y	2 y	
Status (mortality rate)	4 deceased, 6 alive	12 deceased, 1 alive	Alive	Alive	Alive	62% (16 deceased, 10 alive)
Age at death	Infancy (1–13 mo), 1 deceased *in utero*	Infancy (4 days to 2 y), 6 y, 37 y				
**Neonatal characteristics**						
Gestational age	NA	Term (5/5)	42 weeks	39 weeks	40 weeks	
Birth weight (kg)	1.48 –3.79 kg	2.4–3.2 kg	2.6 kg	2.7 kg	3.47 kg	
Birth length (cm)	37–48 cm	42–44 cm	NA	NA	47 cm (3rd centile)	
IUGR	+ (7/10)	+(4/5)	+	–	–	75% (12/16)
Microcephaly/OFC (cm)	+ (5/10)/31–35 cm	−(0/5)/33.4–41.6 cm	NA	NA	−/38.1 cm (98th centile)	31% (5/16)
**Current data**						
Short stature	+ (6/7)	+ (11/12)	+	+	+	90% (18/20)
Height (cm) at last measurement	145.2 (P10 at 13 y)	145 (V-4 at 37 y)	142 (−5.0 SD)	134.3 (−6.3 SD)	74.4 (−4.5 SD)	
Weight (kg) at last measurement	45.4 (P10 at 13 y)	21.5 (< 0.4th centile) (V-4 at 15 y)	45.9 (−1.6 SD)	45 (−1.5 SD)	8.5 (−4.8 SD)	
OFC (cm)	49–58.9 cm	NA	53.2 cm	NA	46.5 cm (13th centile)	
Distinctive facial features	+ (8/10)	+ (2/2, IV-6 and V-11)	+	+	+	87% (13/15)
**CNS**						
Global developmental delay	+ (10/10)	+ (11/11)	+	+	+	100% (24/24)
Hypotonia	+ (7/9)	+ (10/11)	+	+	+	87% (20/23)
CNS anomaly	+ (10/10)	+ (6/6)	+	+	- Microhemorrhage only	95% (18/19)
Seizures/Epilepsy	+ (4/7)	+ (4/11)	+	+	-	48% (10/21)
**Cardiovascular**						
Congenital cardiac anomaly	+ (6/10) ASD, VSD, DORV, PA, PS	+ (9/12) ASD, PDA, VSD, PS	–	–	+ TOF	64% (16/25)
**Genitourinary anomaly**						
Hypospadias	+ (5/10)	+ (6/6)	–	–	+	63%(12/19)
Cryptorchidism	+ (5/10)	+ (2/6)	–	–	+	42%(8/19)
Renal anomaly	+ (4/10) Hydronephrosis, duplex kidney	+ (2/6) Hydronephrosis	–	–	–	32% (6/19)
**GI problems**						
Feeding difficulty	+ (6/9) GERD, NG tube	+ (11/11) GERD, NG tube, PEG (VI-6)	+ NG tube	–	+ GERD	83% (19/23)
**Others**						
Polydactyly	+ (4/10)	−(0/13)	–	–	–	15% (4/26)
Hirsutism	+ (4/9)	−(0/13)	–	–	–	16% (5/25)
**Laboratory data abnormality**						
	Thrombocytopenia, leukopenia, transaminitis, mild elevation in CK levels, low folate levels	–	–	–	Low growth hormone, undetectable IGF1 and IGFBP3	20% (5/24)

*LINKED, LINKage-specific-deubiquitylation-deficiency-induced embryonic defects; IUGR, intrauterine growth retardation; CNS, central nervous system; OFC, occipitofrontal circumference; ASD, atrial septal defect; VSD, ventricular septal defect; DORV, double outlet right ventricle; PA, pulmonary atresia; PS, pulmonary stenosis; PDA, patent ductus arteriosus; TOF, tetralogy of Fallot; GI, gastrointestinal; GERD, gastroesophageal reflux disease; NG, nasogastric, PEG, percutaneous endoscopic gastrostomy; CK, creatine kinase; IGF1, insulin-like growth factor-1; IGFBP3, insulin-like growth factor binding protein-3; NA, not available/not applicable.*

Symptoms frequently observed were global developmental delay (100%), abnormal head imaging (95%), hypotonia (87%), short stature (90%), distinctive facial features (87%), and feeding diffilulty in infancy (83%). Congenital heart malformations (64%) and hypospadias (63%) are also relatively common. Of note, the distinctive facial features of our three patients are very similar to those of cases in the previous reports mentioned above (Beck et al., 2021; Tripolszki et al., 2021). In the clinical setting, the facial gestalt approach may be useful for diagnosing this syndrome. In addition, the younger brother was unable to walk, whereas the older brother was able to walk with support. This finding suggest that differences in severity can occur even with the same variant, which was also mentioned in a previous report (Tripolszki et al., 2021).

The phenotypes of our patients may help to clarify the genotype–phenotype correlation of this new X-linked disorder and allow for better understanding of the long-term prognosis of these patients. In conclusion, we described three living patients with *OTUD5* missense variants associated with LINKED syndrome. LINKED syndrome may have a longer life expectancy than previously thought.

## Web Resources Used for This Study

CADD: https://cadd.gs.washington.edu/snv

Clustal Omega: https://www.ebi.ac.uk/Tools/msa/clustalo/

ExAC: http://exac.broadinstitute.org/

gnomAD: http://gnomad.broadinstitute.org/

HGMD: http://www.hgmd.cf.ac.uk/ac/index

MutationTaster: http://www.mutationtaster.org/

NHLBI Exome Variant Server (ESP6500): http://evs.gs.washington.edu/EVS/

OMIM: https://www.omim.org/

PolyPhen-2: http://genetics.bwh.harvard.edu/pph2/

Protein Data Bank: http://www.wwpdb.org/

SIFT: http://sift.jcvi.org/

## Data Availability Statement

The original contributions presented in the study are included in the article/supplementary material, further inquiries can be directed to the corresponding author/s.

## Ethics Statement

The studies involving human participants were reviewed and approved by The Institutional Review Board of Yokohama City University School of Medicine and Baylor College of Medicine. Written informed consent was obtained from the individual(s) for the publication of any potentially identifiable images or data included in this article.

## Author Contributions

KS performed the genetic analysis, interpreted the data, and wrote the manuscript. TF recruited the patients, performed the clinical evaluation, and wrote the manuscript. DS and AN evaluated the patients and contributed to the manuscript revising. TS and KO conducted protein structure analysis. AH-G helped to organize patient data. SL designed the research protocol. MSA consented patient. HS ordered genetic testing. PL and HD performed genetic testing. TM, SM, and MA contributed to genetic data analysis. TO, NMi, and NMa conducted and supervised the study, evaluated the data, and wrote the manuscript. All authors contributed to the article and approved the submitted version.

## Conflict of Interest

The authors declare that the research was conducted in the absence of any commercial or financial relationships that could be construed as a potential conflict of interest.

## Footnotes

1 https://www.baylorgenetics.com/

## Abbreviations

DUBs: deubiquitinating enzymes
LINKED: LINKage-specific-deubiquitylation-deficiency-induced embryonic defects
MRI: magnetic resonance imaging
OTU: ovarian tumor
SD: standard deviation
XLID: X-linked intellectual disability
WES: whole-exome sequencing.

## References

[B1] Asahina M. Endoh Y. Matsubayashi T. Hirano K. Fukuda T. Ogata T. (2016). Genomewide array comparative genomic hybridization in 55 Japanese normokaryotypic patients with non-syndromic intellectual disability. *J. Pediatr. Neurol. Disord.* 2:108. 10.4172/2572-5203.1000108

[B2] Basar M. A. Beck D. B. Werner A. (2020). Deubiquitylases in developmental ubiquitin signaling and congenital diseases. *Cell Death Differ.* 10.1038/s41418-020-00697-5 [Epub ahead of print]. 33335288 PMC7862630

[B3] Beck D. B. Basar M. A. Asmar A. J. Thompson J. Oda H. Uehara D. T. (2021). Linkage-specific deubiquitylation by OTUD5 defines an embryonic pathway intolerant to genomic variation. *Sci. Adv.* 7:eabe2116. 10.1126/sciadv.abe2116 PMC7817106 33523931

[B4] Collett-Solberg P. F. Liu G. T. Satin-Smith M. Katz L. L. Moshang T. Jr. (1998). Pseudopapilledema and congenital disc anomalies in growth hormone deficiency. *J. Pediatr. Endocrinol. Metab.* 11 261–265. 10.1515/jpem.1998.11.2.261 9642641

[B5] Huang O. W. Ma X. Yin J. Flinders J. Maurer T. Kayagaki N. (2012). Phosphorylation-dependent activity of the deubiquitinase DUBA. *Nat. Struct. Mol. Biol.* 19 171–175. 10.1038/nsmb.2206 22245969

[B6] Komander D. Clague M. J. Urbe S. (2009). Breaking the chains: structure and function of the deubiquitinases. *Nat. Rev. Mol. Cell Biol.* 10 550–563. 10.1038/nrm2731 19626045

[B7] Kulathu Y. Komander D. (2012). Atypical ubiquitylation – the unexplored world of polyubiquitin beyond Lys48 and Lys63 linkages. *Nat. Rev. Mol. Cell Biol.* 13 508–523. 10.1038/nrm3394 22820888

[B8] Li Q. Wang K. (2017). InterVar: clinical interpretation of genetic variants by the 2015 ACMG-AMP guidelines. *Am. J. Hum. Genet.* 100 267–280. 10.1016/j.ajhg.2017.01.004 28132688 PMC5294755

[B9] Lubs H. A. Stevenson R. E. Schwartz C. E. (2012). Fragile X and X-linked intellectual disability: four decades of discovery. *Am. J. Hum. Genet.* 90 579–590. 10.1016/j.ajhg.2012.02.018 22482801 PMC3322227

[B10] Mevissen T. E. Hospenthal M. K. Geurink P. P. Elliott P. R. Akutsu M. Arnaudo N. (2013). OTU deubiquitinases reveal mechanisms of linkage specificity and enable ubiquitin chain restriction analysis. *Cell* 154 169–184. 10.1016/j.cell.2013.05.046 23827681 PMC3705208

[B11] Miyake N. Takahashi H. Nakamura K. Isidor B. Hiraki Y. Koshimizu E. (2020). Gain-of-function MN1 truncation variants cause a recognizable syndrome with craniofacial and brain abnormalities. *Am. J. Hum. Genet.* 106 13–25. 10.1016/j.ajhg.2019.11.011 31839203 PMC7042485

[B12] Muthusamy B. Selvan L. D. N. Nguyen T. T. Manoj J. Stawiski E. W. Jaiswal B. S. (2017). Next-generation sequencing reveals novel mutations in X-linked intellectual disability. *OMICS* 21 295–303. 10.1089/omi.2017.0009 28481730 PMC5586158

[B13] Neri G. Schwartz C. E. Lubs H. A. Stevenson R. E. (2018). X-linked intellectual disability update 2017. *Am. J. Med. Genet. A* 176 1375–1388. 10.1002/ajmg.a.38710 29696803 PMC6049830

[B14] Richards S. Aziz N. Bale S. Bick D. Das S. Gastier-Foster J. (2015). Standards and guidelines for the interpretation of sequence variants: a joint consensus recommendation of the American College of Medical Genetics and Genomics and the Association for Molecular Pathology. *Genet. Med.* 17 405–424. 10.1038/gim.2015.30 25741868 PMC4544753

[B15] Rosenfeld J. A. Drautz J. M. Clericuzio C. L. Cushing T. Raskin S. Martin J. (2011). Deletions and duplications of developmental pathway genes in 5q31 contribute to abnormal phenotypes. *Am. J. Med. Genet. A* 155A 1906–1916. 10.1002/ajmg.a.34100 21744490

[B16] Sobreira N. Schiettecatte F. Valle D. Hamosh A. (2015). GeneMatcher: a matching tool for connecting investigators with an interest in the same gene. *Hum. Mutat.* 36 928–930. 10.1002/humu.22844 26220891 PMC4833888

[B17] Tripolszki K. Sasaki E. Hotakainen R. Kassim A. H. Pereira C. Rolfs A. (2021). An X-linked syndrome with severe neurodevelopmental delay, hydrocephalus, and early lethality caused by a missense variation in the OTUD5 gene. *Clin. Genet.* 99 303–308. 10.1111/cge.13873 33131077

[B18] Tzschach A. Grasshoff U. Beck-Woedl S. Dufke C. Bauer C. Kehrer M. (2015). Next-generation sequencing in X-linked intellectual disability. *Eur. J. Hum. Genet.* 23 1513–1518. 10.1038/ejhg.2015.5 25649377 PMC4613482

[B19] Wiszniewska J. Bi W. Shaw C. Stankiewicz P. Kang S. H. Pursley A. N. (2014). Combined array CGH plus SNP genome analyses in a single assay for optimized clinical testing. *Eur. J. Hum. Genet.* 22 79–87. 10.1038/ejhg.2013.77 23695279 PMC3865406

[B20] Yang Y. Muzny D. M. Reid J. G. Bainbridge M. N. Willis A. Ward P. A. (2013). Clinical whole-exome sequencing for the diagnosis of mendelian disorders. *N. Engl. J. Med.* 369 1502–1511. 10.1056/NEJMoa1306555 24088041 PMC4211433

[B21] Yang Y. Muzny D. M. Xia F. Niu Z. Person R. Ding Y. (2014). Molecular findings among patients referred for clinical whole-exome sequencing. *JAMA* 312 1870–1879. 10.1001/jama.2014.14601 25326635 PMC4326249

